# Cumulative receiver operating characteristics for analyzing interaction between tissue visfatin and clinicopathologic factors in breast cancer progression

**DOI:** 10.1186/s12935-018-0517-z

**Published:** 2018-02-09

**Authors:** Sin-Hua Moi, Yi-Chen Lee, Li-Yeh Chuang, Shyng-Shiou F. Yuan, Fu Ou-Yang, Ming-Feng Hou, Cheng-Hong Yang, Hsueh-Wei Chang

**Affiliations:** 1 Department of Electronic Engineering, National Kaohsiung University of Applied Sciences, Kaohsiung, Taiwan; 2 Translational Research Center, Kaohsiung Medical University Hospital, Kaohsiung Medical University, Kaohsiung, Taiwan; 3 Department of Anatomy, School of Medicine, College of Medicine, Kaohsiung Medical University, Kaohsiung, Taiwan; 4 Department of Chemical Engineering & Institute of Biotechnology and Chemical Engineering, I-Shou University, Kaohsiung, Taiwan; 5 Cancer Center, Kaohsiung Medical University Hospital, Kaohsiung Medical University, Kaohsiung, Taiwan; 6 Division of Breast Surgery and Department of Surgery, Kaohsiung Medical University Hospital, Kaohsiung, Taiwan; 7 Institute of Clinical Medicine, Kaohsiung Medical University, Kaohsiung, Taiwan; 8 Kaohsiung Municipal Hsiao-Kang Hospital, Kaohsiung Medical University, Kaohsiung, Taiwan; 9 Graduate Institute of Clinical Medicine, Kaohsiung Medical University, Kaohsiung, Taiwan; 10 Institute of Medical Science and Technology, National Sun Yat-sen University, Kaohsiung, Taiwan; 11 Department of Medical Research, Kaohsiung Medical University Hospital, Kaohsiung, Taiwan; 12 Department of Biomedical Science and Environmental Biology, Kaohsiung Medical University, Kaohsiung, Taiwan

**Keywords:** Visfatin, Progression, Breast cancer, Receiver operating characteristic (ROC), Interaction

## Abstract

**Background:**

Visfatin has been reported to be associated with breast cancer progression, but the interaction between the visfatin and clinicopathologic factors in breast cancer progression status requires further investigation. To address this problem, it is better to simultaneously consider multiple factors in sensitivity and specificity assays.

**Methods:**

In this study, a dataset for 105 breast cancer patients (84 disease-free and 21 progressing) were chosen. Individual and cumulative receiver operating characteristics (ROC) were used to analyze the impact of each factor along with interaction effects.

**Results:**

In individual ROC analysis, only 3 of 13 factors showed better performance for area under curve (AUC), i.e., AUC > 7 for hormone therapy (HT), tissue visfatin, and lymph node (LN) metastasis. Under our proposed scoring system, the cumulative ROC analysis provides higher AUC performance (0.746–0.886) than individual ROC analysis in predicting breast cancer progression. Considering the interaction between these factors, a minimum of six factors, including HT, tissue visfatin, LN metastasis, tumor stage, age, and tumor size, were identified as being highly interactive and associated with breast cancer progression, providing potential and optimal discriminators for predicting breast cancer progression.

**Conclusion:**

Taken together, the cumulative ROC analysis provides better prediction for breast cancer progression than individual ROC analysis.

**Electronic supplementary material:**

The online version of this article (10.1186/s12935-018-0517-z) contains supplementary material, which is available to authorized users.

## Background

A high occurrence of ongoing breast cancer progression and metastasis events influences quality of life and survival in breast cancer patients, providing a rationale for an in-depth analysis of the interaction among clinicopathologic factors [1], such as age, family history, genetic specificity, hormonal status and lifestyle [2–7]. Early diagnosis of breast cancer followed by local and/or systematic treatments produces high survival rates [8]. However, recurrence or metastasis of breast cancer is sometimes unavoidable and reduces survival rate [6].

Clinicopathologic factors such as age, tumor size, stage, nodal status, hormone receptor (HR) status (estrogen receptor (ER), progesterone receptor (PR), and human epidermal growth factor receptor 2 (HER2)) are widely considered in risk-adapted therapy decisions and as prognostic indicators for breast cancer progression [6, 7]. However, these factors are generally taken individually without considering possible mutual interactions.

Visfatin (nicotinamide phosphoribosyltransferase or NAMPT) [9] plays an important role in regulating metabolism, inflammation, and carcinogenesis [10, 11]. Visfatin was also reported to overexpress in many types of cancer, such as colorectal [12], prostatic [13], gastric [14], esophageal [15], and breast cancer [16–20]. Therefore, visfatin is considered to be an oncoprotein. Our previous study [21] reported that visfatin was highly expressed in breast cancer tissues and is associated with tumor size, ER negativity (−), PR (−), and poor disease-free/overall survival as well as low recurrence rates for hormone therapy. The correlation between visfatin and several clinicopathologic factors were analyzed individually [21], but possible interaction between visfatin and breast cancer progression-associated clinicopathologic factors has not been investigated. Moreover, the specificity and sensitivity of visfatin as an independent prognosis predictor for breast cancer has not been addressed.

Receiver operating characteristic (ROC) analysis is widely used to evaluate diagnostic test performance for predicting dichotomous results by comparing sensitivity versus specificity [22, 23]. ROC analysis is also used to evaluate the accuracy of various biomarkers for diagnosis, prognosis and survival predictions for cancer patients [24–31]. Recently, ROC analysis has been improved by simultaneously considering multiple factors in terms of cumulative ROC analysis [32–34].

Previously statistical analysis approaches for breast cancer prognosis typically depend on regression models, including logistic regression and the Cox proportional regression method. ROC analysis could provide another strategy for breast cancer prognosis based on sensitivity and specificity. In addition, possible interaction between visfatin and clinicopathologic factors in breast cancer progression has not been investigated. Thus, this study aims to evaluate the interaction effects of clinicopathologic factors in breast cancer progression and identifies high risk factors through cumulative ROC analysis.

## Methods

### Dataset information

The dataset was derived from our previous work [21] with Institutional Review Board (IRB) approval (KMUH-IRB-980567) and informed consent. A total of 105 female breast cancer patients treated with surgery but without radiotherapy or chemotherapy were enrolled at the Department of Surgery, Kaohsiung Medical University Hospital during 2003–2008. Data including visfatin levels and clinicopathological factors is downloadable at https://wp.kmu.edu.tw/changhw/files/2017/05/ROC_tissue-visfatin_dataset.xlsx.

### Grouping for breast cancer progression

Breast cancer patients were classified into progressing and disease-free groups. The progressing group included patients with local/regional recurrence or distant metastasis of breast cancer along with those did not survive the follow-up period. The disease-free group included patients who remained disease-free for 60 months following initial diagnosis and who survived the follow-up period.

### Clinicopathologic factors

As standard prognostic indicators for breast cancer progression [6, 7], we included 13 clinicopathologic factors: Visfatin positive cells (%), tumor stage, grade and size, age, body mass index (BMI), lymph node (LN) metastasis, radiotherapy (RT), chemotherapy (CT), hormone therapy (HT), and breast cancer molecular makers (ER, PR, and HER2).

### Individual ROC analysis

Clinicopathologic factors were dichotomized by ROC curve analysis. The area under the ROC curve (AUC) is used to calculate the accuracy of dichotomous results for predicting the risk of breast cancer progression. A higher AUC value represents a better prognosis prediction for breast cancer progression.

### Cumulative ROC analysis

To score cumulative ROC analysis, the top-ranked factors are ranked according to the AUC of individual ROC analysis. This system transforms individual ROC analysis data into dichotomous data for cumulative ROC analysis. In brief, the cutoff point for each clinicopathologic factor was determined from its individual ROC curve. Values of each factor either above or below its the cutoff point were respectively assigned scores of 1 and 0. All data were transformed into this scoring system for further cumulative ROC analysis.

Different factors were chosen to calculate their cumulative AUC values. This cumulative analysis began with selecting the first two factors with the top AUC values for an individual ROC. Other factors were then tested as the third one and the cumulative AUC value was calculated. Only the factor that provided the highest cumulative AUC value was kept. This process was repeated for other factor combinations. Accordingly, different numbers of clinicopathologic factors that generated the largest cumulative AUC value were selected to investigate the joint effects of clinicopathologic factors associated with breast cancer progression.

### Statistical analyses

Table 1 summarizes the distribution of clinicopathologic factors between the disease-free and the progressing groups. The difference between both groups was estimated using the Fisher’s exact test or χ^2^ test. The dichotomous analysis for single and combinational clinicopathologic factors were determined by individual and cumulative ROC as mentioned above (2.3 and 2.4). Combining different factors, the likelihood ratio (LR) [23] was chosen to evaluate disease-free status in patients. LR+, i.e., sensitivity/(1 − specificity) [23], is the ratio of the probability of a positive test in the progressing patients to that of disease-free patients. In contrast, LR−, i.e., 1 − sensitivity)/specificity [23], is the ratio of the probability of a negative test in the progressing patients to that of disease-free patients. All statistical calculations were analyzed by STATA version 11.0.

**Table 1:** Clinicopathologic factors of breast cancer patients in disease-free and progressed statuses. Dataset was retrieved from our previous study [21]

Factors	Disease-free (n = 84)		Progressed^a^ (n = 21)		*P*
	n	%	n	%	
Tissue visfatin					*<* *0.001*
≤ 50%	51	60.7	4	19.0	
> 50%	33	39.3	17	81.0	
Stage					*<* *0.001*
I, II	73	86.9	10	47.6	
III, IV	11	13.1	11	52.4	
Grade					0.669
1, 2	60	71.4	14	66.7	
3	24	28.6	7	33.3	
Age (years)					*0.002*
< 50	51	60.7	5	23.8	
≥ 50	33	39.3	16	76.2	
BMI (kg/m^2^)					0.195
< 24	53	63.1	10	47.6	
≥ 24	31	36.9	11	52.4	
Tumor size (cm)					*0.007*
< 2	44	52.4	4	19.0	
≥ 2	40	47.6	17	81.0	
LN metastasis					*<* *0.001*
Negative	59	70.2	6	28.6	
Positive	25	29.8	15	71.4	
ER					0.051
Positive	59	70.2	10	47.6	
Negative	25	29.8	11	52.4	
PR					0.139
Positive	51	60.7	9	42.9	
Negative	33	39.3	12	57.1	
HER2 status					0.417
Positive	32	38.1	6	28.6	
Negative	52	61.9	15	71.4	
Triple negative					*0.031*
No	70	83.3	13	61.9	
Yes	14	16.7	8	38.1	
RT					0.487
Yes	51	60.7	11	52.4	
No	33	39.3	10	47.6	
CT					0.557
Yes	66	78.6	18	85.7	
No	18	21.4	3	14.3	
HT					*<* *0.001*
Yes	60	71.4	6	28.6	
No	24	28.6	15	71.4	

*BMI* body mass index, *LN* lymph node, *ER* estrogen receptor, *PR* progesterone receptor, *HER2* human epidermal growth factor receptor 2, *RT* radiotherapy, *CT* chemotherapy, *HT* hormone therapy

^a^Progressed patients including patients with breast cancer recurrence or patients expired during 5-year follow-up

## Results

### Clinicopathologic characteristics and progression of breast cancer

Disease progression developed in 21 (20.0%) of the 105 breast cancer patients tracked within 5-year follow-up. Table 1 summarizes the clinicopathologic characteristics of breast cancer patients in disease-free and progressing statuses. Compared to the disease-free patients, the progressing patients had a significantly higher proportion of tissue visfatin > 50% (81.0%), stage III/IV (52.4%), grade 3 (33.3%), age ≥ 52 years (76.2%), tumor size ≥ 2 cm (81.0%), LN metastasis (71.4%), triple negative subtype (38.1%), and hormone therapy (71.4%).

### AUC for individual clinicopathologic factors for breast cancer progression

Table 2 presents the dichotomous results for the various single clinicopathologic factors listed in Table 1. Hormone therapy (AUC = 0.7143), tissue visfatin (AUC = 0.7083), and LN metastasis (AUC = 0.7083) show acceptable performance in the AUC results (≥ 0.7) under single factor analysis, indicating these clinicopathologic factors are acceptable prognosis factors for breast cancer progression. The AUC values for other factors were less than 0.7.

**Table 2:** Top-ranked results of individual ROC in predicting breast cancer progression. Dataset was retrieved from our previous study [21]

Factors	Individual AUC	High risk	Low risk	Sensitivity (%)	Specificity (%)
HT	0.7143	No	Yes	71.43	71.43
Tissue visfatin	0.7083	> 50%	≤ 50%	80.95	60.71
LN metastasis	0.7083	Positive	Negative	71.43	70.24
Stage	0.6964	III, IV	I, II	52.38	86.90
Age	0.6845	≥ 50 years	< 50 years	76.19	60.71
Tumor size (cm)	0.6667	≥ 2 cm	< 2 cm	80.95	52.38
ER	0.6131	Negative	Positive	52.38	70.24
PR	0.5893	Negative	Positive	57.14	60.71
BMI	0.5774	≥ 24	< 24	52.38	63.10
HER2	0.5476	Negative	Positive	71.43	38.10
RT	0.5417	No	Yes	47.62	60.71
Grade	0.5238	3	1, 2	33.33	71.43
CT	0.4643	No	Yes	14.29	78.57

*AUC* Area under receiver operating characteristic. Others were shown in Table 1

### Cumulative ROC analysis of breast cancer progression

Our proposed cumulative ROC analysis scoring system was used to determine the combined effects of tissue visfatin and these clinicopathologic factors. As shown in Table 3, the cumulative AUC value for the combinations of cumulative top-ranked clinicopathologic factors was calculated using the AUC value for different factor combinations, where the factors were added individually in descending order of individual AUC value (Table 2). The scoring system obtained the highest AUC value (0.886) for the 6 cumulative top-ranked factors combination, including HT, tissue visfatin, LN metastasis, stage, age, and tumor size. AUC values increased from 0.764 to 0.886 for the cumulative top-ranked clinicopathologic factors, but decreased slightly after combining 7 or more cumulative top-ranked factors. Therefore, 6 dichotomized clinicopathologic factors yielded the best prognosis prediction for breast cancer progression.

**Table 3:** Top-ranked results of cumulative ROC in predicting breast cancer progression^a^. Dataset was retrieved from our previous study [21]

Cumulative top-ranked factors	Factors	Cumulative AUC	95% CI
2	HT and tissue visfatin	0.764	0.64–0.89
3	Above factors plus LN metastasis	0.827	0.73–0.93
4	Above factors plus stage	0.854	0.77–0.93
5	Above factors plus age	0.886	0.82–0.95
*6*	*Above factors plus tumor size*	*0.886*	*0.82*–*0.95*
7	Above factors plus ER	0.880	0.81–0.95
8	Above factors plus PR	0.863	0.78–0.94
9	Above factors plus BMI	0.861	0.77–0.95
10	Above factors plus HER2	0.863	0.78–0.95
11	Above factors plus RT	0.858	0.77–0.94
12	Above factors plus Grade	0.847	0.76–0.93
13	Above factors plus CT	0.841	0.76–0.93

Abbreviations: See Table 1

^a^Progressed patients including patients with breast cancer recurrence or patients expired during 5-year follow-up

### Cumulative ROC analysis performance for predicting breast cancer progression

According to the cumulative top-ranked clinicopathologic factors scoring results, 6 selected dichotomized clinicopathologic factors provided scores ranging from 0 to 6 (Table 4). Both scores 1 and 2 have 100% sensitivity (i.e., all progressing patients are correctly classified). However, score 2 has a higher specificity compared to score 1 (45.24% vs 14.29%), indicating that score 2 can predict disease-free patients more precisely. Score 4 has the best result, with a correctly classification of 83.81% for disease progression with the best combination of sensitivity (76.19%) and specificity (85.71%) as well as good LR performance (LR+ 5.33 and LR− 0.28).

**Table 4:** Performance of scores from cumulative ROC analysis in predicting breast cancer progression. Dataset was retrieved from our previous study [21]

Scores of dichotomized factors^a^	Sensitivity (%)	Specificity (%)	Sensitivity + specificity (%)	Correctly classified (%)	LR+	LR−
1	100.00	14.29	114.29	31.43	1.17	0.00
2	100.00	45.24	145.24	56.19	1.83	0.00
3	95.24	65.48	160.72	71.43	2.76	0.07
4	76.19	85.71	161.90	83.81	5.33	0.28
5	42.86	94.05	136.91	83.81	7.20	0.61
6	19.05	97.62	116.67	81.90	8.00	0.83

*LR+* Likelihood ratio for a positive test result, *LR*− Likelihood ratio for a negative test result

^a^The number of dichotomized factors was the cumulative effects of the various clinicopathologic factors from Table 3, including HT, tissue visfatin, LN metastasis, stage, age, and tumor size

### Distribution of scores and selected factors associated with breast cancer progression

Because score 1 has low sensitivity (Table 4) and may provide little contribution to predicting breast cancer progression, we started the distribution analysis from score 2 (Table 5). Scores 5 and 6 have similar specificity levels (94.05 and 97.26%) but score 6 has relatively poor sensitivity (19.05%). Thus, score 6 provides little contribution to predicting breast cancer progression and we combined score 6 with score 5 for distribution analysis. Accordingly, the final prognosis scores ranged from ≤ 2 to ≥ 5.

**Table 5:** Distribution for scores and selected factors associated with breast cancer progression. Dataset was retrieved from our previous study [21]

Score^a^	Total	Non-HT		Tissue visfatin > 50%		LN metastasis		Stage III, IV		Age > 50 years		Tumor size ≥ 2 cm	
		(n = 39)		(n = 50)		(n = 40)		(n = 22)		(n = 49)		(n = 57)	
	n^b^	n^c^	%	n^c^	%	n^c^	%	n^c^	%	n^c^	%	n^c^	%
≤ 2	56	–		1	1.8	2	3.6	–		1	1.8	–	
3	21	2	9.5	2	9.5	2	9.52	–		2	9.52	2	9.52
4	14	5	35.7	5	35.7	3	21.4	4	28.6	5	35.7	6	42.9
≥ 5	14	8	57.1	9	64.3	8	57.1	7	50.0	8	57.1	9	64.3

^a^Cumulative score (Table 4) representing the number of risk properties of the selected clinicopathologic factors (HT, tissue visfatin, LN metastasis, stage, age, and tumor size) in patients

^b^n is the number of all breast cancer patients with disease-free or progressed conditions

^c^n is the number of breast cancer progressed patients

As shown in Table 5, 56 (53.3%) of the 105 patients scored ≤ 2, but only 2 (3.6%) were progressing patients with LN metastasis, 1 (1.8%) had tissue visfatin > 50%, and 1 (1.8%) was age > 50 years. A total of 21 (20.0%) patients scored 3, 2 (9.5%) of which were progressing patients with non-HT (no receiving HT), tissue visfatin > 50%, LN metastasis, age > 50 years, and tumor size ≥ 2 cm. In patients who scored 4, about half were progressing patients with tumor size ≥ 2 cm (42.9%; 6/14), followed by non-HT, tissue visfatin > 50%, and age > 50 years (35.7%; 5/14). A total of 9 (64.3%) of 14 progressing patients with a score of ≥ 5 had tissue visfatin > 50% and tumor size ≥ 2 cm, followed by non-HT, LN metastasis and age > 50 years (57.1%; 8/14).

## Discussion

We previously reported that the correlation between tissue visfatin and several individual clinicopathologic factors [21], but the specificity and sensitivity, along with mutual interactions, were not investigated for breast cancer progression. In the current study, individual ROC analysis was used to evaluate the specificity and sensitivity performance of these clinicopathologic factors associated with breast cancer progression. However, as shown in Table 2, aside from HT, tissue visfatin and LN metastasis, the other clinicopathologic factors displayed weak ROC performance (individual AUC < 0.7), and contributed less impact to breast cancer progression. These results suggest that many single factors may have a differential impact on breast cancer progression. Moreover, the possible interactions between tissue visfatin and clinicopathologic factors were not examined by individual ROC analysis.

A similar effect (Additional file 1) was found in univariate logistic regression results, but multivariate analysis produced inconsistent results, except for LN metastasis. According to the individual ROC results (Table 2), the individual AUC of LN metastasis is 0.7083, with a cumulative AUC combining HT, tissue visfatin, LN metastasis, stage, and age, and the tumor size is 0.886. Although multivariate logistic regression analysis presents a novel approach to breast cancer diagnosis, the ROC analysis approach based on sensitivity and specificity also provides acceptable results.

Cumulative ROC analysis has recently been applied in many research fields [32–37]. Six of 25 cephalometric measurements were selected by cumulative ROC analysis to optimize the determination of surgical or nonsurgical treatment for skeletal Class III malocclusions [32]. Cumulative ROC analysis has also been applied in cancer association studies, identifying cumulative risks for five SNPs for association with papillary thyroid carcinoma [33]. Thus, cumulative ROC can effectively identify joint effects of multiple disease- or gene-associated factors.

Similarly, our study developed a scoring system for multiple factors-based cumulative ROC analysis and identified a minimum of 6 clinicopathologic factors for predicting breast cancer progression: HT, tissue visfatin, LN metastasis, tumor stage, age, and tumor size (Table 3). Only 4 of these 6 factors provided acceptable sensitivity, specificity, and classification accuracy for predicting breast cancer progression through cumulative ROC analysis with a high AUC score of 0.886 (Table 4). These results suggest that the cumulative ROC analysis strategy can be effectively used to identify the joint effect of multiple factors for breast cancer progression.

Recently, interactions between genes and physical activities has been emphasized [38–40], with a particular focus on genetic polymorphisms. In the current study, we focus on the genetic expression of tissue visfatin in terms of protein level in tissue histochemistry. Cumulative ROC analysis shows tissue visfatin levels can combine with common clinicopathologic factors to provide an analysis of the interaction between breast tumor biomarkers and clinicopathologic factors.

Among these interactions, HT is the best single predictor for breast cancer progression (Table 2). Clinical studies have suggested that breast cancer patients may benefit from adjuvant HT [41–43]. Tissue visfatin has also been reported to be an important biomarker for breast cancer progression [21]. In the current study, HT and tissue visfatin were shown to have a strong joint association with breast cancer progression. In addition, tumor size and lymph node metastasis are common prognosis factors for both breast cancer progression [44, 45] and Nottingham prognostic index [45]. Advanced stage (III/IV) breast cancer is typically characterized by greater tumor size, lymph node invasion, pathological change in the surrounding tissue, or metastases in distant organs. Advanced stage tumor burden is usually associated with a poor disease prognosis in breast cancer [46–48]. Furthermore, age has been suggested to be an important risk factor for breast cancer, as inferred from the association with age-related tissue microenvironment alteration [49]. Moreover, therapeutic impact differs with age [50, 51]. Hence, breast cancer progression is significantly associated with tumor characteristics and aging [48], but how this age-dependency increases in disease progression remains unclear. Moreover, the proportion of progressing patients with 6 selected high-risk clinicopathologic factors (No-HT, tissue visfatin > 50%, LN metastasis, tumor stage III/IV, age > 50 years, and tumor size ≥ 2 cm) increased with cumulative score, indicating that these selected high-risk clinicopathologic factors were highly associated with breast cancer progression. Accordingly, these factors exhibit a high degree of mutual interactivity in cumulative ROC analysis.

Although our study did not consider the follow-up interval for breast cancer progression, our proposed strategy for cumulative ROC analysis shows the joint effect of clinicopathologic factors for predicting breast cancer progression in terms of sensitivity and specificity for disease prediction. Furthermore, the proposed scoring system was based on the highest AUC performance from cumulative ROC analysis and did not account for the association of low AUC clinicopathologic factors effects on breast cancer progression.

The present study is subject to several limitations. No follow-up intervals are included and the cumulated ROC approach is mainly affected by the AUC ranking results, which might restrict exhaustive search for all possible combinations of complex interactions. For example, although ER, PR and Her2 status were reported to be highly associated with breast cancer progression, the cumulated AUC ranking results for the combined interactions still excluded these 3 factors. In addition, we did not include molecular subtype (TNBC, luminal A, luminal B and Her 2 enriched). Moreover, the visfatin level was examined from the tumor specimens of breast cancer patients, indicating the visfatin level required invasive procedures. Further examination of visfatin level through the secretary samples such as blood should be considered.

## Conclusions

The current study examined the impact of 13 clinicopathologic factors for predicting breast cancer progression. Under our proposed scoring system, the cumulative ROC analysis provides better AUC performance than individual ROC analysis in predicting breast cancer progression. Analysis of factor interaction identified a minimum of 6 factors which display a high degree of mutual interaction and association with breast cancer progression: HT, tissue visfatin, LN metastasis, tumor stage, age, and tumor size.

## Additional file

**Additional file 1.** Logistic regression analysis of progression risk factors in breast cancer. This table provides the univariate and multivariate logistic regression analysis of risk factor associated with breast cancer progression.

## Abbreviations

ROC: cumulative receiver operating characteristic
AUC: area under curve
HT: hormone therapy
LN: lymph node
ER: estrogen receptor
PR: progesterone receptor
HER2: human epidermal growth factor receptor 2
NAMPT: nicotinamide phosphoribosyltransferase
BMI: body mass index
CT: chemotherapy
LR: likelihood ratio
