# Supplementary material for: Cumulative receiver operating characteristics for analyzing interaction between tissue visfatin and clinicopathologic factors in breast cancer progression
Source: Cancer Cell Int. 2018 Feb 9;18:19. doi: 10.1186/s12935-018-0517-z (PMC5807850; doi:10.1186/s12935-018-0517-z)
Supplement: Supplementary file 1 — Additional file 1. Logistic regression analysis of progression risk factors in breast cancer. This table provides the univariate and multivariate logistic regression analysis of risk factor associated with breast cancer progression. [file 12935_2018_517_MOESM1_ESM.docx]

Additional Table 1. Logistic regression analysis of risk factor associated with breast cancer progression.

| Variable | Comparison | Univariate | | |  | Multivariate | | |
| --- | --- | --- | --- | --- | --- | --- | --- | --- |
|  |  | OR | 95% CI | P |  | OR | 95% CI | P |
| Visfatin | > 50% vs. ≤ 50% | **6.57** | **2.03-21.25** | **0.002** |  | 3.24 | 0.71-14.76 | 0.129 |
| Stage | III,IV vs. I,II | **7.30** | **2.52-21.19** | **<0.001** |  | 2.12 | 0.39-11.61 | 0.385 |
| Grade | 3 vs. 1,2 | 1.25 | 0.45-3.48 | 0.669 |  | 0.87 | 0.22-3.50 | 0.846 |
| Age | ≥ 50 yrs vs. < 50 yrs | **4.95** | **1.65-14.79** | **0.004** |  | 4.01 | 0.93-17.34 | 0.063 |
| BMI | ≥ 24 vs. < 24 | 1.88 | 0.72-4.93 | 0.199 |  | 1.18 | 0.28-4.92 | 0.824 |
| Tumor size (cm) | ≥ 2 cm vs. < 2 cm | **4.68** | **1.45-15.07** | **0.010** |  | 1.67 | 0.37-7.47 | 0.501 |
| LN metastasis | Positive vs. Negative | **5.90** | **2.05-16.96** | **0.001** |  | 9.07 | **1.23-66.59** | **0.030** |
| ER | Negative vs. Positive | 2.60 | 0.98-6.89 | 0.055 |  | 0.97 | 0.07-13.34 | 0.984 |
| PR | Negative vs. Positive | 2.06 | 0.78-5.43 | 0.144 |  | 2.27 | 0.31-16.81 | 0.421 |
| HER2 status | Negative vs. Positive | 1.54 | 0.54-4.37 | 0.419 |  | 2.20 | 0.53-9.23 | 0.280 |
| RT | No vs. Yes | 1.40 | 0.54-3.68 | 0.488 |  | 2.24 | 0.43-11.78 | 0.339 |
| CT | No vs. Yes | 0.61 | 0.16-2.31 | 0.468 |  | 0.38 | 0.04-3.75 | 0.407 |
| HT | No vs. Yes | **6.25** | **2.17-18.01** | **0.001** |  | 2.32 | 0.25-21.29 | 0.457 |
